# Comparison of the efficacy of neuromodulation techniques based on corticospinal excitability in improving exercise-induced fatigue in athletes: a systematic review and network meta-analysis

**DOI:** 10.3389/fpsyg.2026.1948789

**Published:** 2026-10-13

**Authors:** Peng Qiurong, Wu Libing, Li Qing

**Affiliations:** 1 Guangdong Ersha Sports Training Center, Guangzhou, China; 2 Department of Social Work, Guangzhou Resettlement Center for Assisted Persons, Guangzhou, China; 3 Affiliated Hospital of Xiangnan University, Chenzhou, China

**Keywords:** acupuncture, athletes, corticospinal excitability, exercise-induced fatigue, network meta-analysis, neuromodulation, transcranial direct current stimulation

## Abstract

**Background:**

Exercise-induced fatigue (EIF) is a core problem in competitive sports, significantly affecting the training quality and performance of athletes. Neuromodulation techniques based on corticospinal excitability regulation—including both central and peripheral stimulation—offer a novel approach to EIF intervention; however, the comparative efficacy of different techniques remains unclear.

**Objective:**

To compare the effects of various neuromodulation techniques on EIF in athletes using a systematic review and network meta-analysis.

**Methods:**

PubMed, Embase, Web of Science, Cochrane Library, China National Knowledge Infrastructure (CNKI), Wanfang Data, and VIP databases were systematically searched for randomized controlled trials comparing neuromodulation techniques with placebo/sham stimulation/blank control from database inception to June 2026. The Cochrane ROB-2 tool was used to assess risk of bias, and the CINeMA framework was applied to evaluate evidence quality. A Bayesian model was employed for network meta-analysis, with SUCRA values calculated to rank efficacy, and meta-regression was used to explore sources of heterogeneity.

**Results:**

A total of 14 RCTs involving 756 athletes were included. The paired meta-analysis showed that neuromodulation techniques significantly reduced BLA levels and alleviated fatigue (MD = −0.47, 95% CI: −0.57, −0.38). Network meta-analysis indicated that TEAS combined with acupuncture ranked first in improving RPE (SUCRA = 99.3%), blood lactate (SUCRA = 96.3%), and BUN (SUCRA = 72.6%). CINeMA evaluation showed that most evidence was of low quality. Meta-regression revealed that the total number of stimulations negatively moderated the effect on blood lactate (*P* = 0.001), and intervention type, risk of bias, and study design significantly influenced BUN outcomes (all *P* < 0.05).

**Conclusion:**

TEAS combined with acupuncture may be associated with the most favorable SUCRA ranking, but this should be interpreted as exploratory for improving EIF in athletes. However, the overall quality of current evidence is low, and SUCRA rankings should be interpreted as exploratory. Future high-quality RCTs are needed to validate these findings and determine the optimal stimulation parameter ranges.

**Systematic review registration:**

https://www.crd.york.ac.uk/PROSPERO/view/CRD420261446854.

## 1 Introduction

Exercise-induced fatigue (EIF) is the most prevalent and inevitable physiological response in competitive sports. It is defined as a transient decline in physical performance capacity resulting from the inability of the body's physiological systems to sustain either a prescribed exercise intensity or a given functional output over time. Epidemiological data from 11 international athletics championships—spanning 59 competition days—revealed that EIF, including associated collapse and performance failure, was the second most frequently reported medical condition after upper respiratory tract infections, accounting for 15.4% of all documented illnesses (Edouard et al., 2019). Furthermore, approximately 24% of athletes engaged in long-term, high-intensity training report persistent fatigue symptoms; notably, those training more than 20 h per week exhibit a 1.69-fold higher relative risk of chronic fatigue compared with their peers training less intensively (Vetter and Symonds, 2010). Critically, EIF not only impairs athletic performance and training efficacy but also elevates the risk of sports-related injuries, disrupts neuromuscular coordination, compromises executive decision-making, and suppresses immune function collectively undermining both competitive advancement and athletic longevity (Meeusen et al., 2013).

Current interventions for EIF primarily encompass conventional recovery strategies, including nutritional support, physical therapy, massage, thermotherapy (e.g., contrast water immersion), and psychological interventions. Yet these approaches predominantly target peripheral mechanisms—such as muscle metabolism and microtrauma repair and demonstrate limited efficacy in modulating central nervous system (CNS)-mediated fatigue pathways (Weerapong et al., 2005). Accumulating evidence indicates that EIF arises not merely from peripheral factors, including energy substrate depletion and metabolite accumulation—but, more fundamentally, from CNS-driven reductions in motor drive, reflected in diminished cortical excitability and attenuated spinal motor neuron output (Gandevia, 2001). Consequently, conventional recovery modalities often fail to rapidly restore CNS function; their effects are typically delayed and insufficient to meet the stringent temporal demands of elite athletes requiring swift, neurophysiologically grounded recovery (Taylor et al., 2016).

In recent years, neuromodulation techniques targeting corticospinal excitability (CSE) have emerged as a promising frontier for intervening in EIF. The corticospinal tract the principal descending pathway linking the primary motor cortex to spinal α-motor neurons serves as the final common neural conduit for voluntary movement. Its excitability level directly governs corticospinal output, synaptic drive to spinal motor neurons, and ultimately, muscle force production. Accumulating evidence demonstrates that EIF is consistently accompanied by a marked reduction in CSE, particularly following prolonged endurance exercise (Gandevia, 2001). Non-invasive brain stimulation modalities—including anodal transcranial direct current stimulation (tDCS) and high-frequency repetitive transcranial magnetic stimulation (rTMS) can augment corticospinal transmission by modulating cortical neuronal membrane potentials or inducing long-term potentiation-like synaptic plasticity, thereby delaying the onset of central fatigue (Angius et al., 2017). A recent meta-analysis confirmed that anodal tDCS significantly enhances attentional performance and cognitive processing speed in athletes while concurrently reducing subjective and objective markers of mental fatigue (Mollazadeh and Ramezanpour, 2026). Complementing central approaches, peripheral neuromodulation techniques,such as acupuncture and transcutaneous electrical acupoint stimulation (TEAS)—have also demonstrated efficacy in potentiating CSE: a meta-analysis of 34 randomized trials involving 1,031 adults reported that both electroacupuncture and manual acupuncture applied to motor-related acupoints significantly increased motor evoked potential (MEP) amplitudes, with standardized effect sizes of 0.53 mV and 0.43 mV, respectively (Liu et al., 2025). Nevertheless, substantial heterogeneity persists across studies: several well-controlled trials failed to detect significant improvements in motor performance following tDCS, and inter-study variability in outcomes remains high (Holgado et al., 2019). Moreover, critical methodological inconsistencies, including lack of consensus on optimal stimulation parameters (intensity, duration, electrode/coil placement), acupoint selection criteria, and treatment course design—undermine evidence comparability and impede clinical translation (Niu et al., 2022).

Although numerous original studies and meta-analyses have independently evaluated the effects of central neuromodulation (e.g., tDCS, rTMS) and peripheral neuromodulation (e.g., acupuncture, TEAS) on EIF, no synthesis has yet integrated these distinct intervention classes within a unified comparative framework (Grandperrin et al., 2020). These techniques differ fundamentally in anatomical target (central cortex vs. peripheral somatosensory afferents), temporal application (pre-exercise prophylaxis vs. post-exercise recovery), and mechanistic pathway (direct modulation of cortical excitability vs. indirect modulation via spinal/supraspinal reflex loops and neurohumoral signaling) (Hutton et al., 2015). Moreover, the evidence base is unevenly distributed across neuromodulation modalities: central techniques—particularly tDCS—have received far more attention than peripheral approaches, with a recent meta-analysis identifying 19 RCTs of tDCS in athletes alone (Holgado et al., 2019), whereas evidence for acupuncture and TEAS remains sparse and fragmented. A systematic review of acupuncture for exercise-induced fatigue identified only 11 RCTs, most conducted in non-athlete or mixed populations (Niu et al., 2022). This imbalance is compounded by substantial methodological heterogeneity across studies, including variability in stimulation parameters, acupoint selection, treatment duration, and outcome assessment timing. Such inconsistencies limit comparability and highlight the need for a unified analytical framework integrating both central and peripheral techniques. To our knowledge, no previous review has systematically compared these two intervention classes within a single network meta-analysis based on their shared mechanistic target of corticospinal excitability. The present study was therefore designed to address this gap.Consequently, their relative efficacy, optimal sequencing, and context-specific indications remain inadequately characterized.

Network meta-analysis (NMA) overcomes this limitation by simultaneously synthesizing both direct (head-to-head) and indirect (via common comparators) evidence, enabling hierarchical ranking of interventions according to effect magnitude and statistical certainty—thereby furnishing clinicians with robust, evidence-based guidance for intervention selection (Borg, 1970). Accordingly, this study employs a rigorous systematic review and network meta-analysis protocol to comparatively evaluate the efficacy of diverse neuromodulation techniques—unified by their shared physiological endpoint of CSE enhancement in alleviating EIF among athletes. The findings aim to deliver high-quality, actionable evidence to inform precision neuromodulation strategies and individualized recovery programming.

## 2 Methods

### 2.1 Protocol and registration

This systematic review was designed and reported in strict accordance with the Preferred Reporting Items for Systematic Reviews and Network Meta-Analyses (PRISMA-NMA) statement and the Cochrane Handbook for Systematic Reviews of Interventions. The study protocol was registered on the PROSPERO Systematic Review Registry (https://www.crd.york.ac.uk/PROSPERO/view/CRD420261446854). Ethical approval and informed patient consent were not required because all data were derived from published studies.

### 2.2 Search strategy and literature screening

The following electronic databases were systematically searched: PubMed, Embase, Web of Science, Cochrane Central Register of Controlled Trials (CENTRAL), China National Knowledge Infrastructure (CNKI), Wanfang Data Knowledge Service Platform, and VIP Chinese Science and Technology Journal Database. The search time limit was from database inception to June 1, 2026. Additionally, reference lists of relevant published reviews and meta-analyses were manually searched to supplement the identification of potential eligible studies. The search terms were combined using medical subject headings (MeSH) and free-text words, and the core search concepts were determined according to the PICOS framework. The search terms included: “athletes/elite athletes/trained individuals,” “exercise-induced fatigue/central fatigue/peripheral fatigue,” “transcranial direct current stimulation/transcranial magnetic stimulation/repetitive transcranial magnetic stimulation/transcutaneous electrical acupoint stimulation/acupuncture,” and “randomized/randomized.”

### 2.3 Inclusion and exclusion criteria

We formulated the inclusion criteria according to the PICOS principles.

**Participants**: Explicitly reported as athletes, regardless of age, sex, race, or sport.

**Intervention**: An intervention program centered on neuromodulation techniques, specifically including: (1) central neuromodulation techniques: transcranial direct current stimulation (tDCS), transcranial alternating current stimulation (tACS), transcranial random noise stimulation (tRNS), repetitive transcranial magnetic stimulation (rTMS), and continuous theta burst stimulation (cTBS); (2) peripheral neuromodulation techniques: acupuncture (manual acupuncture/electroacupuncture), transcutaneous electrical acupoint stimulation (TEAS), and auricular point sticking/auricular point magnetotherapy (AMT). Stimulation parameters (current intensity, frequency, area, duration) and intervention cycles were not restricted. It is important to emphasize that the definition of network nodes was based on the complete intervention protocol actually implemented in each study. Combined interventions were treated as unique intervention nodes within the same network comparison; their effect sizes reflect the comprehensive efficacy of the complete regimen, rather than the independent contribution of any single intervention component.

**Comparator**: Included sham stimulation, sham acupuncture, or blank control. These conditions are not physiologically equivalent: sham procedures may induce mild placebo effects, whereas blank control provides none. To address this heterogeneity, all control conditions were collapsed into a single “placebo” node for the primary analysis, consistent with common practice in neuromodulation network meta-analyses. A sensitivity analysis excluding blank-control studies was planned but could not be performed with adequate statistical power due to the limited number of studies.

**Outcomes**: Including Rating of Perceived Exertion (RPE) (Borg, 1970): a standardized scale for athletes to self-evaluate exercise intensity or fatigue according to their own feelings. Higher scores indicate greater subjective effort. Blood lactate (BLA): a core objective index for evaluating peripheral fatigue and exercise intensity. Higher post-exercise blood lactate concentrations indicate greater anaerobic metabolic burden and deeper fatigue. Blood urea nitrogen (BUN): objectively reflects athletes' tolerance to training load and the degree of fatigue. Higher BUN levels indicate more pronounced fatigue.It should be clarified that the included trials did not directly measure corticospinal excitability (CSE). Rather, the CSE-based framework serves as a conceptual rationale for integrating central and peripheral neuromodulation techniques within a unified comparative network. The primary outcomes of this review (RPE, BLA, and BUN) are indirect indicators of exercise-induced fatigue. The mechanistic link between neuromodulation and CSE enhancement is inferred from previous literature, not directly demonstrated in the included studies. Future studies should incorporate direct CSE measurements as primary outcomes to validate the proposed mechanism.

**Study type**: Randomized controlled trial (RCT), including both parallel and crossover designs.

#### 2.3.2 Exclusion criteria

Non-randomized controlled trials (e.g., self-controlled studies, non-randomized controlled trials, case reports, reviews, conference abstracts, and animal experiments); subjects who were non-athletes or non-regular trainees; interventions that did not conform to the definition of neuromodulation techniques (e.g., pure physical therapy, nutritional supplements, psychological interventions, etc.); studies with inaccessible complete data or data unsuitable for meta-analysis; and duplicate publications (the most complete data or earliest published version were retained).

### 2.4 Data extraction

Two researchers independently screened the literature and extracted the data. The extracted information included: first author, publication year, country, sample size, age, sex ratio, years of training, type of exercise program, specific parameters of intervention measures (stimulation type, intensity, frequency, location, single-session duration, total number of interventions, and cycle), details of control measures, and changes in baseline and final values of outcome indicators (Mean ± SD). If original literature data were missing or incompletely reported, the corresponding author was contacted by email for supplementary information. Disagreements were resolved by discussion or consultation with the third researcher. Two RCTs with crossover designs were included in this study. According to the Cochrane Handbook, mean differences (MD) and standard errors were extracted directly for studies reporting paired data. If only the overall mean of each stage was provided, the MD between the intervention and control terminal values was used as the effect size, and the standard error of the change value was estimated through the standard deviation (SD) reported in the original study and a default correlation coefficient (*r* = 0.5). To control for carryover effects, only cross-phase data were extracted, and washout period data were excluded. Crossover studies used the same MD effect size index as parallel studies in the network meta-analysis, but their weights were calculated based on the paired sample size rather than the independent sample size between groups.

### 2.5 Risk of bias and quality of evidence assessment

Two researchers independently evaluated the methodological quality of the included RCTs using the Cochrane Risk of Bias Tool version 2.0 (RoB 2.0) (Higgins et al., 2024). Evaluation domains included: (1) randomization process; (2) deviations from intended interventions; (3) missing outcome data; (4) outcome measurement; and (5) selective reporting of outcomes. Each study was judged as having “low risk of bias,” “some concerns,” or “high risk of bias.” In case of disagreement, adjudication was discussed with a third investigator. The CINeMA (Nikolakopoulou et al., 2020) system was used to rate the quality of network meta-analysis evidence (Liu et al., 2025), considering five degradation factors: risk of bias, inconsistency, indirectness, imprecision, and publication bias. The final quality of evidence was classified as “high,” “moderate,” “low,” or “very low.”

### 2.6 Effect size calculation

Effect sizes for this meta-analysis were mean difference (MD) and its 95% Bayesian credible interval (CrI). Because the measurement units of the same outcome index were largely consistent across different studies, MD was selected as the effect size. If the original study did not provide the standard deviation (SD) of the change value after intervention, the standard error, 95% CrI, *t*-value, or *p*-value were calculated according to the methods recommended by the Cochrane Handbook. The baseline-to-endpoint correlation coefficient was set at 0.5 to calculate the standard deviation of change. This assumption is based on the moderate correlation between repeated measures, which is widely accepted in the literature, and can balance the potential variation between the two measurements while ensuring the conservatism of the results.

### 2.7 Statistical analysis

#### 2.7.1 Pairwise meta-analysis

When the number of original studies directly comparing an intervention with the same control was ≥3, a random-effects model was used for pairwise meta-analysis. Effect sizes were pooled as mean differences (MD) with 95% confidence intervals. Heterogeneity was assessed using the *I*^2^ statistic: low heterogeneity was defined as *I*^2^ < 25%, moderate heterogeneity as 25%−50%, and high heterogeneity as > 50%. Publication bias was evaluated by funnel plot symmetry and Egger's regression test.

#### 2.7.2 Network meta-analysis

Before conducting the network meta-analysis, the transitivity assumption was evaluated by comparing the inclusion and exclusion criteria, baseline population characteristics, and intervention definitions across studies. Network evidence plots were used to visually display direct and indirect comparisons between different neuromodulation techniques. A Bayesian framework was used for network meta-analysis, implemented using the “gemtc” and “BUGSnet” packages in R software. Markov chain Monte Carlo (MCMC) simulation was run to generate four Markov chains simultaneously, with different initial values selected in random states. The model was iterated for 50,000 times, with the first 10,000 iterations discarded as the burn-in period to eliminate the influence of initial values. Brooks-Gelman-Rubin diagnostics were used to evaluate model convergence. Global inconsistency was evaluated by comparing the deviance information criterion (DIC) and residual deviance between the consistency and inconsistency models. The node-splitting method was used to detect inconsistency between direct and indirect effects within the network framework (*P* < 0.05 was considered inconsistent). The surface under the cumulative ranking curve (SUCRA) was used to show the ranking probability of each intervention. SUCRA ranged from 0 to 100%, with higher values indicating better effects.

#### 2.7.3 Meta-regression

To explore potential effect modifiers that might influence the intervention effect, the following covariates were prespecified for network meta-regression analysis: continuous variables—total number of stimulations, Sample size, single-session duration (minutes), intervention period (weeks), and average age (years); categorical variables, intervention type (central vs. peripheral), study design (parallel vs. crossover), and overall risk of bias (low risk vs. some concerns). All continuous variables were centered before being included in the regression model. A shared regression coefficient model was assumed, meaning that covariates had the same direction of influence on the effect of each intervention.

#### 2.7.4 Assessment of publication bias

For outcome indicators with more than 10 included studies, comparison-adjusted funnel plots were drawn to evaluate small-sample effects and publication bias, and Egger's test was used for statistical inference.

### 2.8 Equity, diversity, and inclusion (EDI) statement

This study was conducted in accordance with the principles of equity, diversity, and inclusion. The research question and intervention strategies examined in this network meta-analysis are relevant to athletes across diverse populations, regardless of sex, race, ethnicity, or socioeconomic background. However, we acknowledge that the included studies predominantly involved male athletes from China and other Asian countries, which may limit the generalizability of our findings to female athletes and other racial/ethnic groups. Additionally, athletes with disabilities, those from low- and middle-income countries, and those participating in non-Olympic sports remain underrepresented in the current evidence base. Future research should prioritize the inclusion of underrepresented populations, including female athletes, athletes with disabilities, and individuals from diverse geographic and cultural backgrounds, to ensure that neuromodulation strategies for EIF are equitable and applicable to all athlete populations.

## 3 Results

### 3.1 Study screening and characteristics

A total of 14 randomized controlled trials (including 2 crossover designs; Chen et al., 2021, 2024; Hu et al., 2024; Lin et al., 2009, 2011; Li et al., 2018; You et al., 2016; Liu, 2021; You et al., 2016; Zhao et al., 2012; Chen, 2014; Li, 2020; Fang, 2025; Lai et al., 2011) involving approximately 756 athletes were included in this study after systematic searching, duplicate removal, and title/abstract screening (Figure 1). The included studies were published between 2009 and 2025. Study sample sizes ranged from 13 to 360. Interventions were divided into two categories: central neuromodulation techniques (tDCS, tPCS, cTBS) and peripheral neuromodulation techniques (acupuncture, TEAS, auricular point sticking, auricular point magnetotherapy, electroacupuncture). In terms of sports, the included studies covered basketball (*n* = 3), football (*n* = 1), rowing (*n* = 1), gymnastics (*n* = 1), and long-distance running (*n* = 1); the remaining six studies involved mixed or unspecified types of regular physical activity. This diversity enhances external generalizability but may also introduce clinical heterogeneity due to differences in fatigue-inducing mechanisms across various sports. Most studies used intervention programs applied immediately after exercise or following fatigue induction, with intervention periods ranging from a single session to 6 weeks (Table 1).

**Figure 1:**
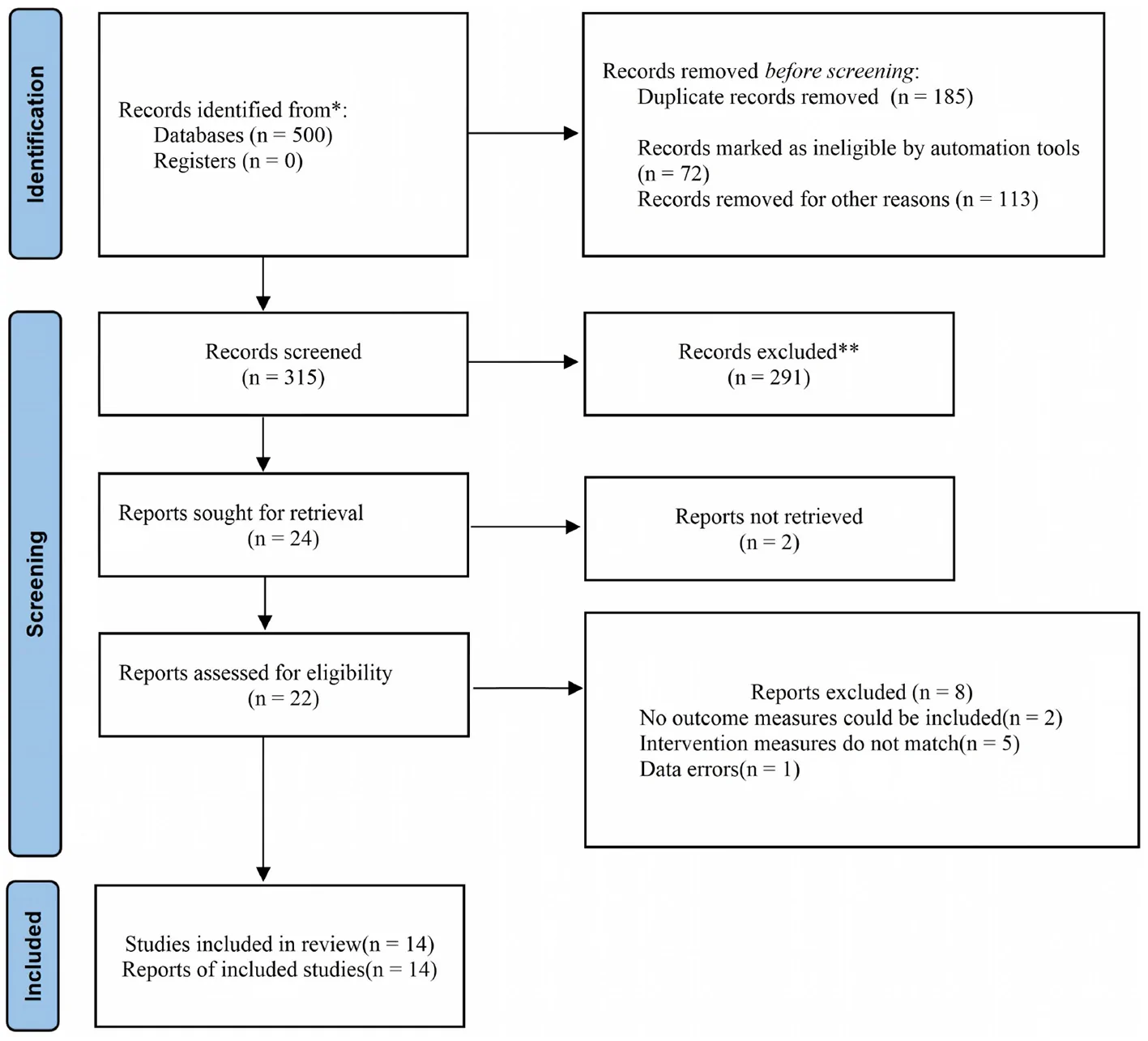
PRISMA flow diagram of the literature screening process and the number of studies included at each stage.

**Table 1:** Basic characteristics of included studies.

References	Year	Experi-mental interven-tion	Control interven-tion	Acting path	Single duration (min)	Number of treat-ments	Study design	Sample size (T/C)	Sex (M/F)	Age (T/C)	Course	Sport type	Outcome measurement timing	Outcomes
Chen et al. (2021)	2021	tDCS	Placebo	Central nervous system	20	1	Crossover	13	13/0	20.3 ± 0.64 (overall)	Single session	Basketball	RPE: Measured during sprinting	RPE
Chen et al. (2024)	2024	cTBS	Placebo	Central nervous system	30	5	Parallel	10/10	20/0	20.85 ± 2.25/20.44 ± 1.74	5 days	NA	RPE: NR (likely measured post-exercise)	RPE
Hu et al. (2024)	2024	Acupuncture	Placebo	Peripheral nervous system	15	1	Parallel	10/10	NR	20.7 ± 1.6 (overall)	Single session	NA	BLA: Measured at baseline, 0 min, and 30 min after fatigue	BLA
Lin et al. (2009)	2009	Acupuncture	Placebo	Peripheral nervous system	NA	1	Parallel	10/10	21.1 ± 0.6	N/A	Single session	Basketball	BLA: Measured at 5th, 30th and 60th min post-exercise	BLA
Lin et al. (2011)	2011	Auricular acupuncture	Placebo	Peripheral nervous system	NA	1	Parallel	12/12	21.2 ± 1.2	N/A	Single session	Basketball	BLA: Measured at 5th, 30th and 60th min post-exercise	BLA
Li et al. (2018)	2018	TEAS	TEAS (no electricity)	Peripheral nervous system	30	29	Parallel	8/8/8	24/0	20.24 ± 3.25 ~ 20.57 ± 3.26	5-6 weeks	Football	BUN: NR	BUN
You et al. (2016)	2016	TEAS	TEAS (no electricity)	Peripheral nervous system	30	12	Parallel	15/15/15	45/0	21.53 ± 1.4 ~ 22.32 ± 1.2	2 weeks	NA	BUN: NR	BUN
Liu (2021)	2021	TEAS	Placebo	Peripheral nervous system	30	12	Parallel	180/180	215/145	21.43 ± 1.36/21.50 ± 1.42	2 weeks	NA	RPE, BLA: NR	RPE, BLA
You et al. (2016)	2016	TEAS	TEAS (no electricity)	Peripheral nervous system	30	12	Parallel	15/15/15	45/0	21.63 ± 1.35 ~ 21.72 ± 1.28	2 weeks	NA	RPE: NR	RPE
Zhao et al. (2012)	2012	TEAS and Acupuncture	Placebo	Peripheral nervous system	40	6	Parallel	10/10	20/0	20 ± 1 (overall)	Single + 5 sessions	run	RPE, BLA, BUN: NR	RPE, BLA, BUN
Chen (2014)	2014	Auricular pressure	Placebo	Peripheral nervous system	NA	14	Parallel	32/30	62/0	20.50 ± 1.05/20.53 ± 1.46	4 weeks	NA	RPE, BUN: NR	RPE, BUN
Li (2020)	2020	Auricular magnetic therapy	Placebo	Peripheral nervous system	NA	42	Parallel	10/10	11/9	19.40 ~ 20.80 (range)	6 weeks	Rowing	RPE, BUN: NR	RPE, BUN
Fang (2025)	2025	tDCS	Placebo	Central nervous system	20	1	Crossover	20	11/9	21.90 ± 2.55 (overall)	Single session	Gymnastics	RPE, BLA: NR	RPE, BLA
Lai et al. (2011)	2011	Acupuncture	Placebo	Peripheral nervous system	30	12	Parallel	33/30	NR	20.15 ± 1.25 ~ 20.53 ± 1.06	2 weeks	NA	BUN: NR	BUN

T, treatment group; C, control group; M, Male; F, Female; tDCS, transcranial direct current stimulation; cTBS, continuous theta burst stimulation; TEAS, transcutaneous electrical acupoint stimulation; RPE, Rating of Perceived Exertion; BLA, Blood lactic acid; BUN, Blood urea nitrogen.

### 3.2 Risk of bias

The Cochrane ROB-2 tool was used to assess the risk of bias of the included studies. In the domain of randomization process, 8 studies (57%) were rated as low risk and 6 studies (43%) as some concerns, mainly due to inadequate description of randomization methods (e.g., only describing “randomized groups” without specifying the specific methods). In the domain of deviations from intended interventions, 9 studies (64%) were rated as low risk and 5 (36%) as some concerns (mainly due to failure to report blinding). In the domain of missing outcome data, 14 studies (100%) were rated as low risk. In the domain of outcome measurement, 14 studies (100%) were rated as low risk. In the domain of selective reporting of outcomes, 14 studies (100%) were rated as low risk. Overall risk of bias: 8 studies (57%) were rated as low risk and 6 (43%) as some concerns (Figure 2).

**Figure 2:**
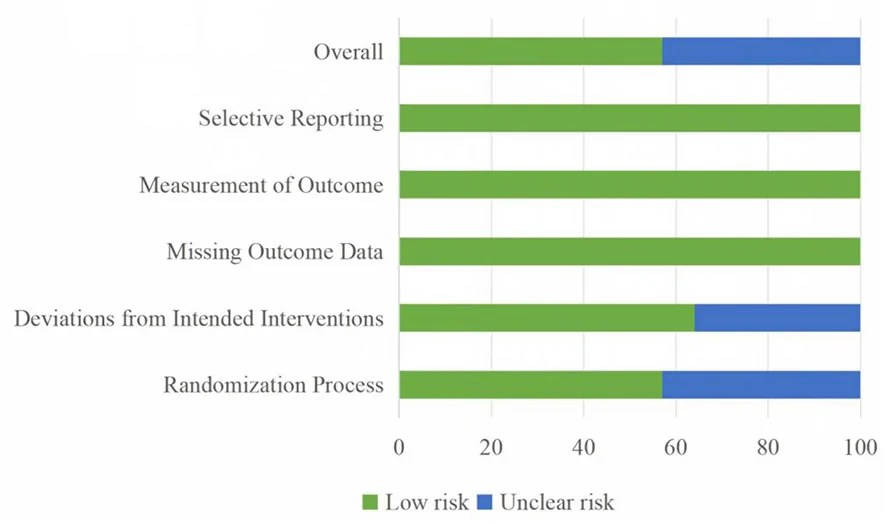
Risk of bias assessment of the included randomized controlled trials using the Cochrane ROB-2 tool, presented as proportions across the five domains and overall judgment.

#### 3.2.3 Assessment of transitivity

The study prespecified the type of exercise program, timing of stimulation, training level, and intervention period as key effect modifiers, and the transitivity assumption was qualitatively evaluated based on the characteristics of the included studies (Table 1). In terms of sports, the included studies covered many categories, including basketball, football, rowing, gymnastics, long-distance running, and regular exercise habits. The diversity of sports was beneficial for external generalizability, but differences in energy metabolism characteristics across sports may act as potential effect modifiers affecting efficacy evaluation. The study covered single-session, short-term repeated (5 days to 2 weeks), and long-term repeated (4 to 6 weeks) intervention cycles, with a relatively scattered distribution. Stimulus timing was the most prominent imbalance in the current network factors—among studies with clear reporting, pre-exercise stimulation, stimulation during exercise, combined stimulation during exercise, and post-exercise/fatigue intervention coexisted, with some Chinese studies not clearly reporting the timing relationship. In conclusion, the current evidence network only partially meets the transitivity assumptions, and the main sources of uncertainty are the uneven distribution of stimulus timing and the clinical heterogeneity of sports types. Therefore, the SUCRA ranking results should be interpreted as exploratory findings rather than definitive conclusions, and the CINeMA evidence quality rating should be considered accordingly.

### 3.3 Meta-analysis

#### 3.3.1 Pairwise meta-analysis

A total of three studies were included in the BLA analysis (Figure 3). The pooled analysis showed that acupuncture could significantly reduce BLA and improve the fatigue state of athletes (MD = −0.47, 95% CI: −0.57, −0.38). Heterogeneity across studies was low (*I*^2^ = 2.1%).

**Figure 3:**
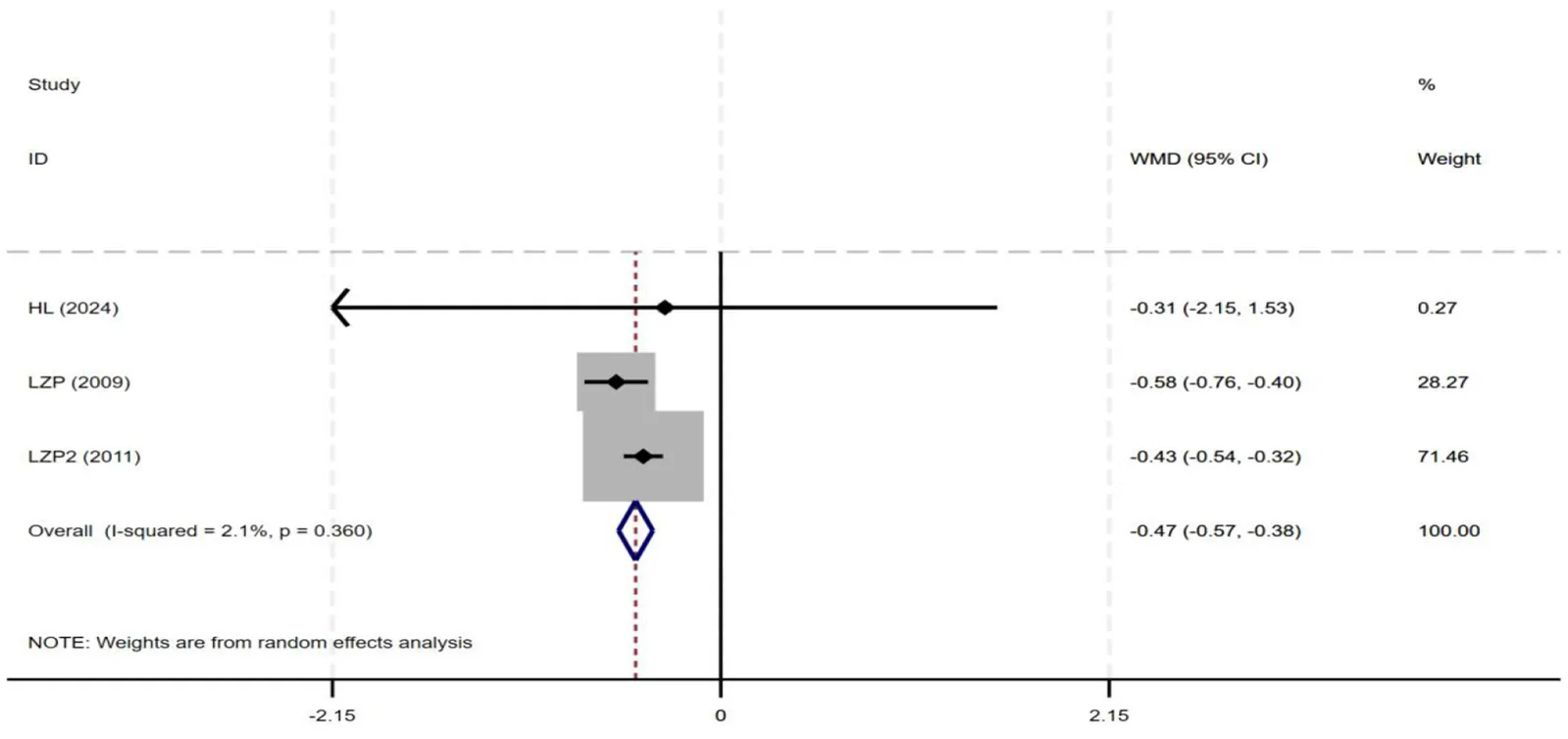
Forest plot of the pairwise meta-analysis comparing acupuncture versus control for blood lactate (BLA).

#### 3.3.2 Results of network meta-analysis

##### 3.3.2.1 Model convergence and fit

A Bayesian random-effects model was used for network meta-analysis. The potential scale reduction factor (PSRF) for all outcome indicators was close to 1.00, indicating good convergence of the Markov chains. The deviance information criterion (DIC) for RPE was 37.73 (19 data points, residual ratio 0.995), for BLA was 28.77 (14 data points, ratio 1.067), and for BUN was 22.49 (12 data points, ratio 0.941), indicating good model fit. Global consistency checks did not reveal significant inconsistency (RPE: *I*^2^ = 5%; BLA: *I*^2^ = 13%; BUN: *I*^2^ = 3%), and the node-splitting method also detected no significant local inconsistency (*P* > 0.05).

##### 3.3.2.2 Rating of perceived exertion (RPE)

A total of eight studies were included, involving eight interventions: AMT, auricular pressure, cTBS, placebo, tDCS, TEAS, TEAS combined with acupuncture, and TEAS (no electricity; Figure 4). Effect sizes compared with placebo: auricular pressure (MD = −5.00, 95% CrI: −6.8, −3.0), cTBS (MD = −5.8, 95% CrI: −8.4, −3.2), TEAS (MD = −6.4, 95% CrI: −7.7, −5.0), TEAS combined with acupuncture (MD = −11.0, 95% CrI: −13.0, −8.0), and TEAS (no electricity; MD = −5.8, 95% CrI: −7.5, −4.0) significantly reduced RPE scores (Figure 5, Supplementary material). SUCRA ranking: TEAS combined with acupuncture had the best effect (SUCRA = 99.3%), followed by TEAS (SUCRA = 79.1%), cTBS (SUCRA = 64.8%), TEAS (without electricity; SUCRA = 64.2%), auricular pressure (SUCRA = 48.4%), AMT (27.6%), placebo (SUCRA = 9.8%), and tDCS (SUCRA = 6.6%; Figure 6, Table 2).

**Figure 4:**
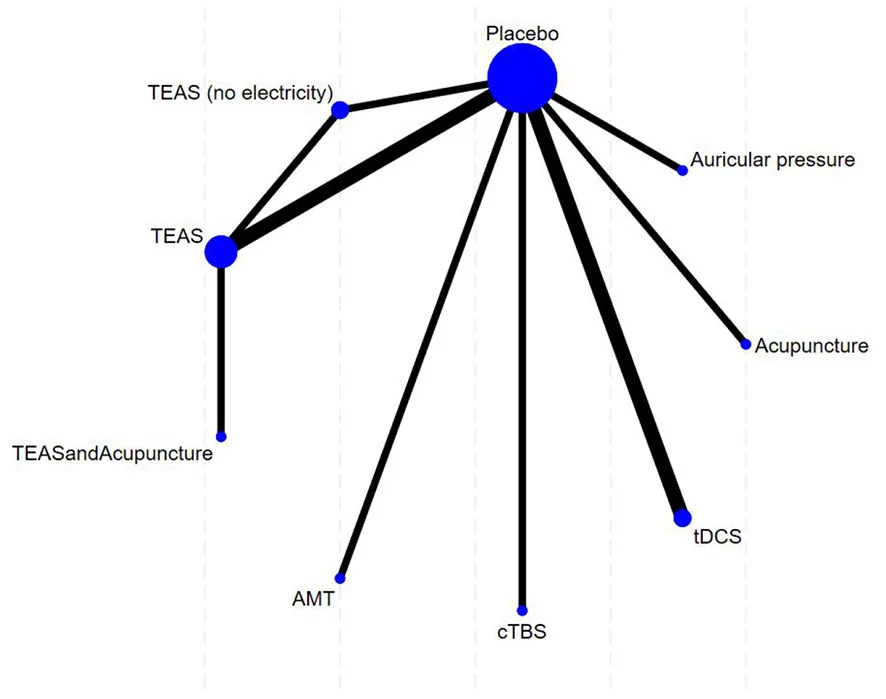
Network evidence plot of all interventions included in the network meta-analysis for the outcome of Rating of Perceived Exertion (RPE).

**Figure 5:**
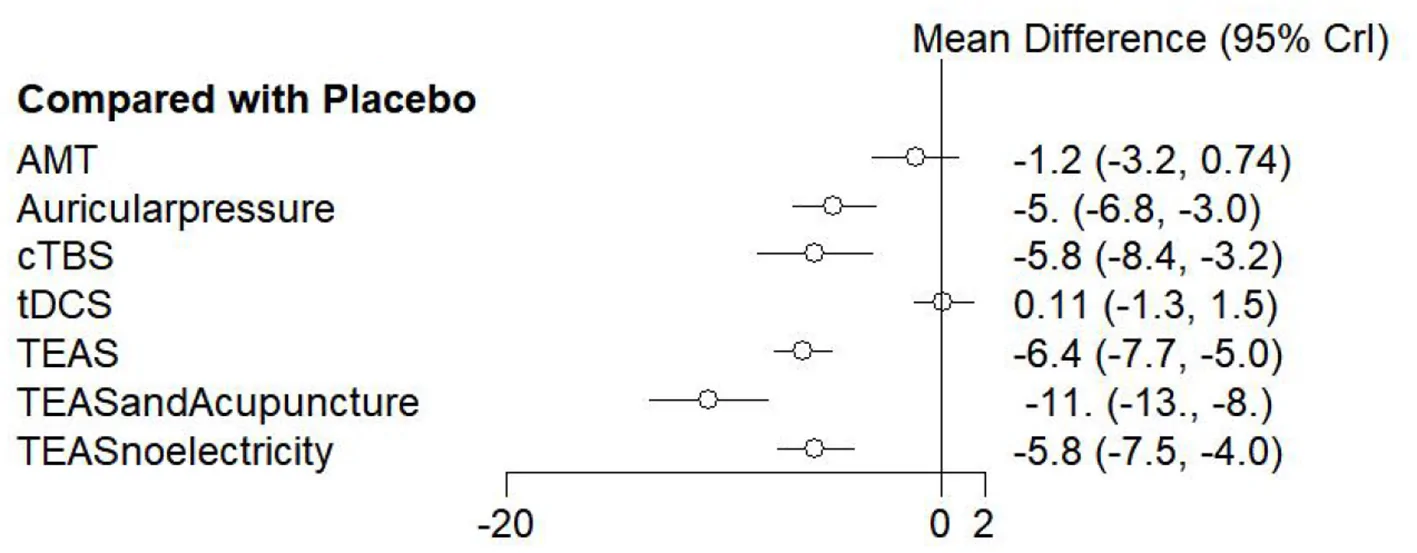
Forest plot of the network meta-analysis results for RPE, showing mean differences with 95% credible intervals for each intervention compared with placebo.

**Figure 6:**
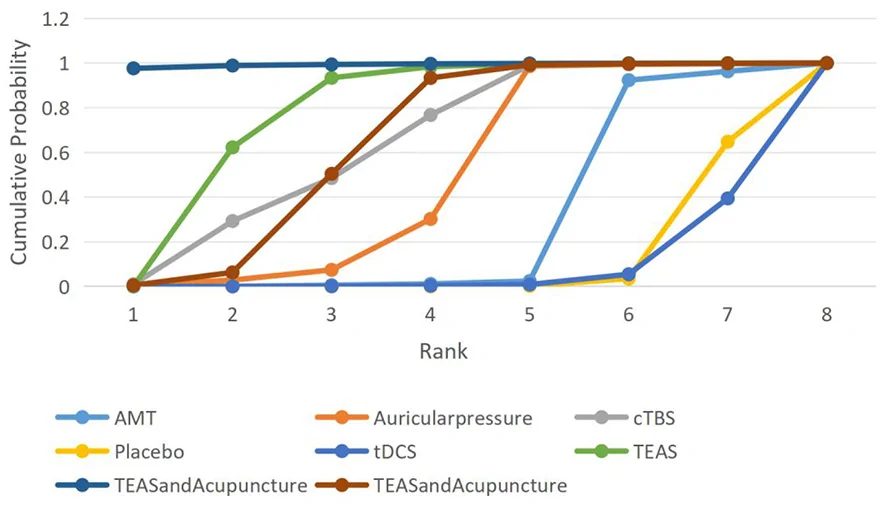
SUCRA (Surface Under the Cumulative Ranking) probability ranking chart for all interventions on RPE.

**Table 2:** SUCRA ranking.

Outcomes	RPE		BLA		BUN	
	SUCRA	Rank	SUCRA	Rank	SUCRA	Rank
AMT	27.6%	5			60.5%	2
Auricular pressure	48.4%	6			38.1%	6
cTBS	64.8%	3				
Placebo	9.8%	7	6.3%	5	16.4%	7
tDCS	6.6%	8	38.2%	3		
TEAS	79.1%	2	74.6%	2	58.4%	3
TEASandAcupuncture	99.3%	1	96.3%	1	72.6%	1
TEAS (no electricity)	64.2%	4			57.1%	4
Acupuncture			34.3%	4	46.6%	5

tDCS, transcranial direct current stimulation; cTBS, continuous theta burst stimulation; TEAS, transcutaneous electrical acupoint stimulation; RPE, Rating of Perceived Exertion; BLA, Blood lactic acid; BUN, Blood urea nitrogen.

##### 3.3.2.3 Blood lactate (BLA)

A total of six studies were included, involving five interventions: acupuncture, placebo, tDCS, TEAS, and TEAS combined with acupuncture (Figure 7). Compared with placebo, TEAS (MD = −2.0, 95% CrI: −3.4, −0.6) and TEAS combined with acupuncture (MD = −3.0, 95% CrI: −5.1, −0.9) both significantly reduced BLA and promoted fatigue reduction (Figure 8, Supplementary material). SUCRA ranking: TEAS combined with acupuncture had the best effect (SUCRA = 96.3%), followed by TEAS (SUCRA = 74.6%), tDCS (SUCRA = 38.2%), acupuncture (SUCRA = 34.3%), and placebo (SUCRA = 6.3%; Figure 9, Table 2).

**Figure 7:**
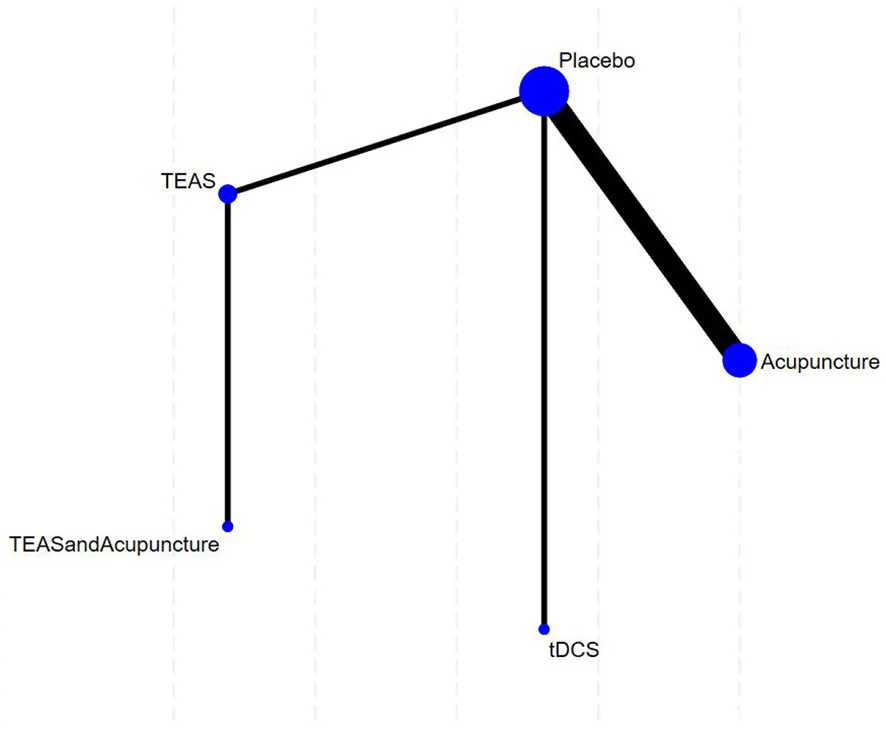
Network evidence plot of all interventions included in the network meta-analysis for the outcome of blood lactate (BLA).

**Figure 8:**
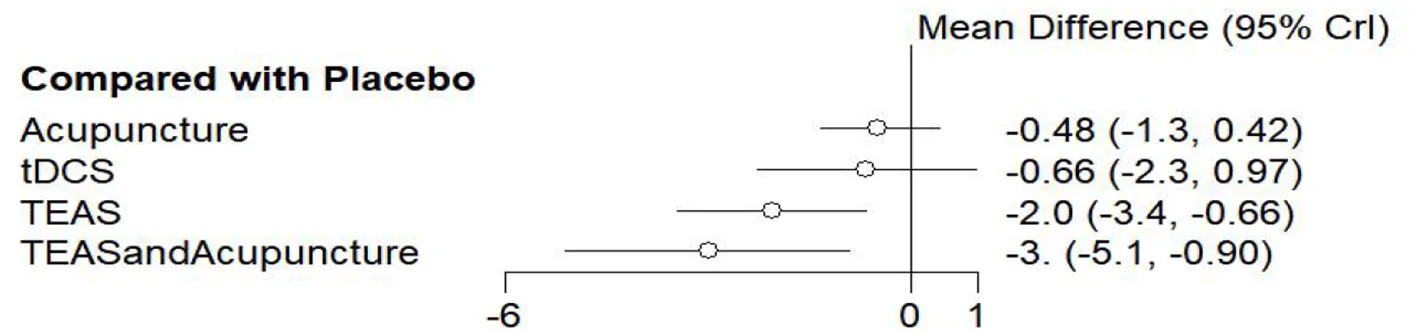
Forest plot of the network meta-analysis results for BLA, showing mean differences with 95% credible intervals for each intervention compared with placebo.

**Figure 9:**
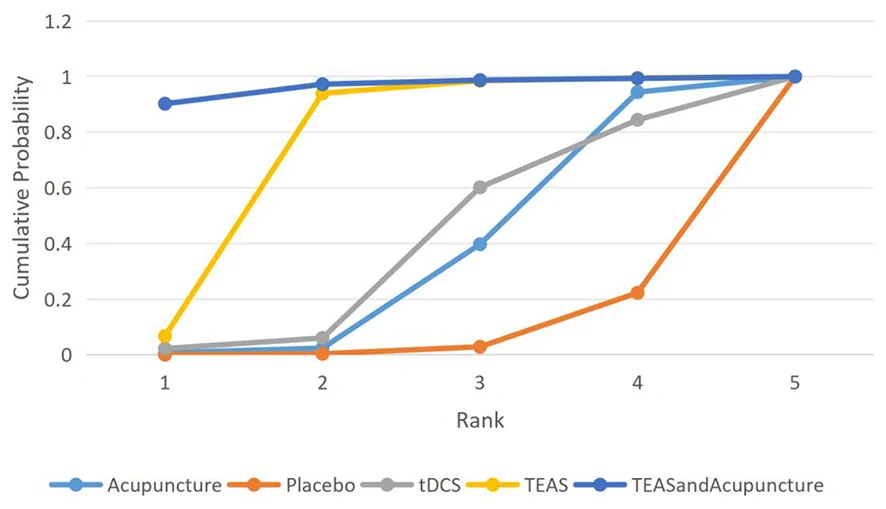
SUCRA probability ranking chart for all interventions on BLA.

##### 3.3.2.4 Blood urea nitrogen (BUN)

A total of six studies with seven interventions were included: acupuncture, AMT, auricular pressure, placebo, TEAS, TEAS (without electricity), and TEAS combined with acupuncture (Figure 10). Compared with placebo, no intervention showed a statistically significant difference (Figure 11). SUCRA ranking: TEAS combined with acupuncture had the best effect (SUCRA = 72.6%), followed by AMT (SUCRA = 60.5%), TEAS (SUCRA = 58.4%), TEAS (no electricity; SUCRA = 57.1%), acupuncture (SUCRA = 46.6%), auricular pressure (SUCRA = 38.1%), and placebo (SUCRA = 16.4%; Figure 12, Table 2). Since the number of included studies was less than 10, publication bias tests were not conducted.

**Figure 10:**
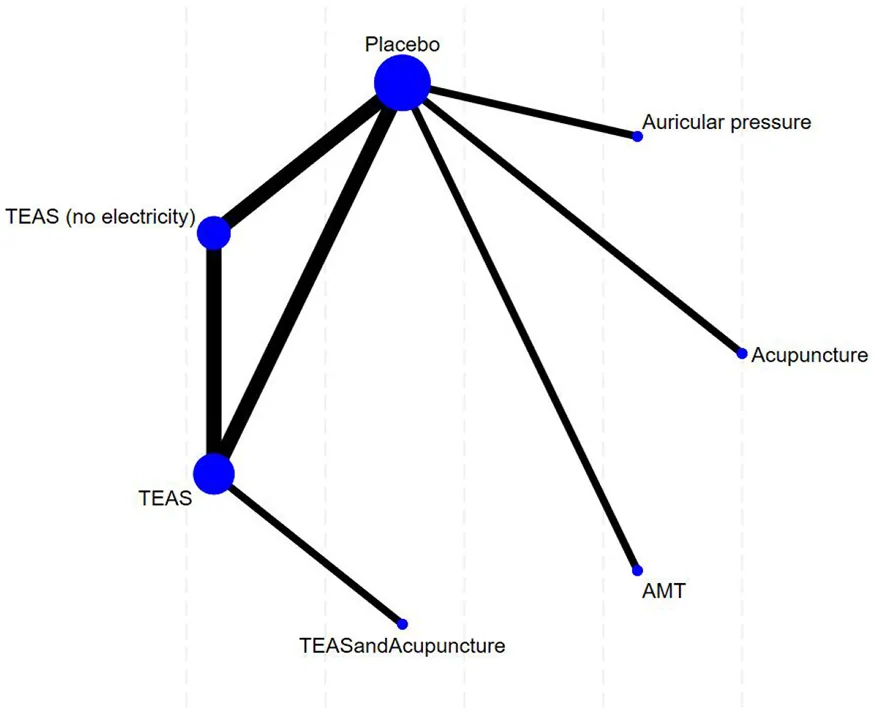
Network evidence plot of all interventions included in the network meta-analysis for the outcome of blood urea nitrogen (BUN).

**Figure 11:**
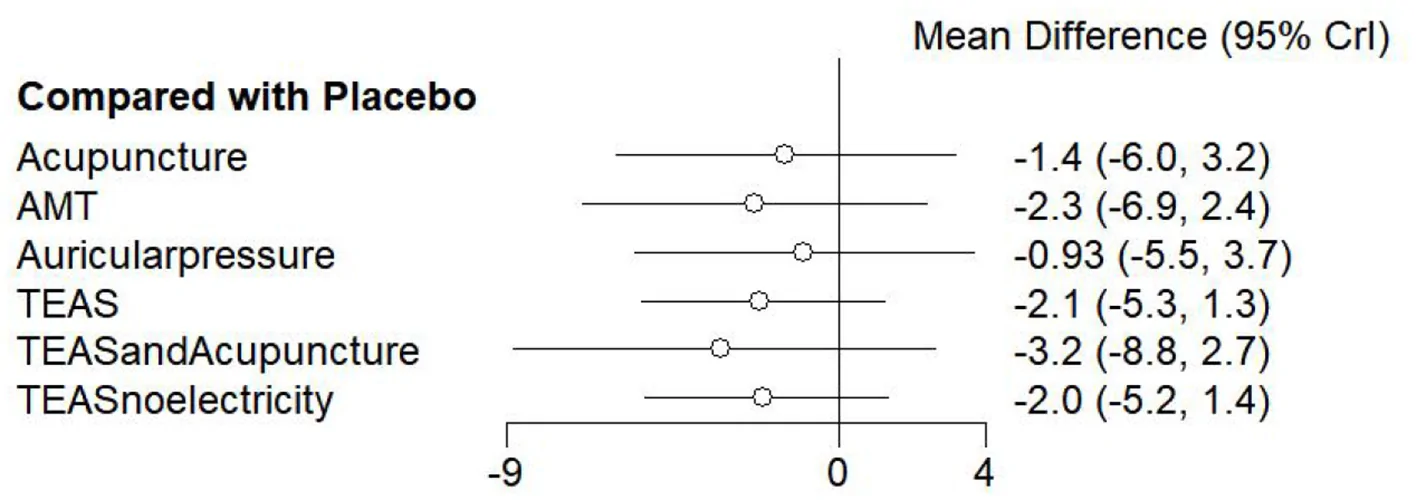
Forest plot of the network meta-analysis results for BUN, showing mean differences with 95% credible intervals for each intervention compared with placebo.

**Figure 12:**
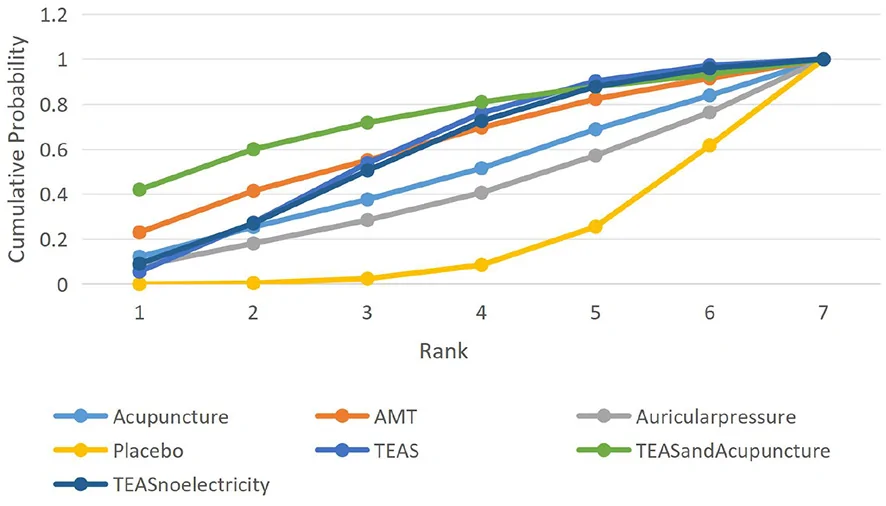
SUCRA probability ranking chart for all interventions on BUN.

It should be emphasized that SUCRA values represent the probability of each intervention ranking first under the available evidence, rather than definitive differences in efficacy. Given the low to very low quality of evidence for most comparisons, the SUCRA rankings should be interpreted as exploratory and hypothesis-generating, not as conclusive rankings of intervention efficacy.

### 3.6 Meta-regression

Regarding blood lactate (BLA), total stimulation frequency significantly negatively moderated the intervention effect (*P* = 0.001), indicating that higher total stimulation counts were associated with weaker improvements in blood lactate levels. Additionally, sample size showed a significant positive moderation on BLA improvement (*P* = 0.01), suggesting that larger studies may report greater effects on blood lactate reduction. For RPE, intervention type showed marginal significance (*P* = 0.06), suggesting central stimulation might be superior to peripheral stimulation; however, sample size did not significantly moderate RPE outcomes (*P* = 0.30). Regarding BUN, intervention type significantly moderated the effect (*P* = 0.03), indicating that central stimulation was more effective than peripheral stimulation; sample size had no significant moderating effect on BUN (*P* = 0.76). Other covariates, including single-stimulation duration, mean age, study design, bias risk, and publication year, did not significantly influence the intervention effects across outcome measures (*P* > 0.05). It should be emphasized that due to the limited number of included studies, this meta-regression analysis lacks sufficient statistical power. The results should therefore be interpreted as exploratory and hypothesis-generating rather than definitive conclusions, and must be cautiously evaluated, not taken as the primary findings of this study (Supplementary materials).

### 3.7 Results of the CINeMA evidence quality evaluation

This study used the CINeMA framework to evaluate the evidence quality of 23 pairwise comparisons (RPE: 9, BUN: 7, BLA: 7) in the network meta-analysis, covering six dimensions: within-study bias, reporting bias, indirectness, imprecision, heterogeneity, and incoherence. Within-study bias, reporting bias, and indirectness were rated as “low risk” for all comparisons, with no downgrading. However, due to the small number of included studies, imprecision for most comparisons was rated as “major concerns,” and most comparisons for RPE and BUN indicators showed concerns regarding heterogeneity and inconsistency, while BLA indicators were rated as “no concerns.” Overall, 7 comparisons for RPE were of very low quality and 2 were of low quality; for BUN, 5 were very low quality and 2 were low quality; for BLA, 4 were very low quality, 1 was low quality, and 2 were moderate quality. A total of 16 indicators (69.6%) were of very low quality, 5 indicators (21.7%) were of low quality, and only 2 indicators (8.7%) were of moderate quality. No high-quality evidence was identified. The main reasons for low evidence quality were imprecision and inconsistency, suggesting that current conclusions need to be interpreted cautiously and further verified by larger, multi-center studies in the future (Table 3).

**Table 3:** CINeMA evidence quality assessment results.

Outcomes	Comparison	Number of studies	Within-study bias	Reporting bias	Indirectness	Imprecision	Heterogeneity	Incoherence	Overall quality
RPE	AMT:Placebo	1	No concerns	Low risk	No concerns	Some concerns	Some concerns	Major concerns	Very Low
	Acupuncture:Placebo	1	No concerns	Low risk	No concerns	No concerns	Major concerns	Major concerns	Very Low
	Auricular pressure:Placebo	1	No concerns	Low risk	No concerns	Some concerns	Some concerns	Major concerns	Very Low
	Placebo:TEAS	2	No concerns	Low risk	No concerns	No concerns	Major concerns	Major concerns	Very Low
	Placebo:TEAS (no electricity)	1	No concerns	Low risk	No concerns	No concerns	Major concerns	No concerns	Low
	Placebo:cTBS	1	No concerns	Low risk	No concerns	Major concerns	No concerns	Major concerns	Very Low
	Placebo:tDCS	2	No concerns	Low risk	No concerns	No concerns	Major concerns	Major concerns	Very Low
	TEAS:TEAS (no electricity)	1	No concerns	Low risk	No concerns	No concerns	Major concerns	No concerns	Low
	TEAS:TEASandAcupuncture	1	No concerns	Low risk	No concerns	Major concerns	No concerns	Major concerns	Very Low
BUN	AMT:Placebo	1	No concerns	Low risk	No concerns	Major concerns	No concerns	Major concerns	Very Low
	Acupuncture:Placebo	1	No concerns	Low risk	No concerns	Major concerns	No concerns	Major concerns	Very Low
	Auricular pressure:Placebo	1	No concerns	Low risk	No concerns	Major concerns	No concerns	Major concerns	Very Low
	Placebo:TEAS	2	No concerns	Low risk	No concerns	Major concerns	No concerns	Major concerns	Very Low
	Placebo:TEAS (no electricity)	1	No concerns	Low risk	No concerns	Major concerns	No concerns	No concerns	Low
	TEAS:TEAS (no electricity)	1	No concerns	Low risk	No concerns	Major concerns	No concerns	No concerns	Low
	TEAS:TEASandAcupuncture	1	No concerns	Low risk	No concerns	Major concerns	No concerns	Major concerns	Very Low
BLA	AMT:Placebo	1	No concerns	Low risk	No concerns	Major concerns	No concerns	Major concerns	Very Low
	Acupuncture:Placebo	1	No concerns	Low risk	No concerns	Major concerns	No concerns	Major concerns	Very Low
	Auricular pressure:Placebo	1	No concerns	Low risk	No concerns	Major concerns	No concerns	Major concerns	Very Low
	Placebo:TEAS	1	No concerns	Low risk	No concerns	Some concerns	No concerns	No concerns	Moderate
	Placebo:TEAS (no electricity)	1	No concerns	Low risk	No concerns	Major concerns	No concerns	No concerns	Low
	TEAS:TEAS (no electricity)	1	No concerns	Low risk	No concerns	No concerns	No concerns	No concerns	Moderate
	TEAS:TEASandAcupuncture	1	No concerns	Low risk	No concerns	Major concerns	No concerns	Major concerns	Very Low

tDCS, transcranial direct current stimulation; cTBS, continuous theta burst stimulation; TEAS, transcutaneous electrical acupoint stimulation; RPE, Rating of Perceived Exertion; BLA, Blood lactic acid; BUN, Blood urea nitrogen.

### 3.8 Assessment of publication bias

Because the number of direct comparison studies included for each outcome indicator in this study was less than 10, according to Cochrane Handbook recommendations and the general standards of previous meta-analysis methodology literature (Higgins et al., 2023), when the number of included studies is less than 10, the statistical power of funnel plot symmetry tests and Egger's regression tests is insufficient to reliably distinguish publication bias from chance variation (Sterne et al., 2011). Therefore, no formal statistical test for publication bias was performed in this study. However, the impact of publication bias on the results was minimized by comprehensively searching multiple Chinese and English databases and manually searching the reference lists of included studies and relevant published reviews.

## 4 Discussion

### 4.1 Summary of evidence

This is the first systematic review and network meta-analysis to comprehensively compare the efficacy of central and peripheral neuromodulation techniques in improving EIF in athletes, based on the common target of corticospinal excitability. A total of 14 randomized controlled trials were included, evaluating the intervention effects of tDCS, tPCS, cTBS, acupuncture, TEAS, auricular point sticking, auricular point magnetotherapy, and electroacupuncture on three fatigue indicators: RPE, BLA, and BUN. Pairwise meta-analysis showed that acupuncture intervention could significantly reduce blood lactate in athletes. The SUCRA ranking results from the network meta-analysis showed that TEAS combined with acupuncture ranked first in improving RPE, BLA, and BUN, demonstrating the best overall efficacy. Among central stimulation techniques, cTBS performed prominently in improving RPE, while tDCS ranked lower across the three outcome indicators. These findings suggest that neuromodulation techniques based on corticospinal excitability modulation, especially combined peripheral and central intervention strategies, have important potential in improving EIF in athletes. It should be noted that the CINeMA evidence quality ratings for all outcome measures in this study were low, and the SUCRA ranking results should be viewed as exploratory findings rather than definitive conclusions. Because most comparisons were based on single studies, network inconsistency could not be fully assessed, and confidence intervals for effect estimates were generally wide; current evidence is insufficient to support any intervention as a definitive clinical recommendation. The SUCRA ranking reflects the probability of being the best treatment given the limited available evidence, rather than the certainty of differences in effectiveness between interventions. Therefore, the application of our findings should be limited to guiding future research rather than directly informing clinical decision-making. The following discussion is based on this premise.

### 4.2 Evidence for efficacy of optimal interventions

This study found that TEAS combined with acupuncture ranked first in improving the three core indicators of EIF, which can be understood from both mechanistic and clinical evidence perspectives. From a mechanistic standpoint, TEAS combined with acupuncture may exert a synergistic effect through a “peripheral–central” dual regulatory pathway. As a traditional body surface stimulation therapy, acupuncture has been shown to enhance corticospinal excitability by activating abundant nerve endings beneath acupoints and transmitting signals through the spinal cord to the cerebral motor cortex. A meta-analysis involving 34 studies and 1,031 adults confirmed that both electroacupuncture and manual acupuncture at motor-related acupoints significantly increased motor evoked potential (MEP) amplitudes, with effect sizes of 0.53 mV and 0.43 mV, respectively (Liu et al., 2025). TEAS, as a transcutaneous electrical acupoint stimulation technique, is essentially an “electrical” modification of traditional acupuncture. By stimulating the nerve terminals beneath acupoints with specific frequencies (2–15 Hz), TEAS can also enhance corticospinal excitability through the peripheral–central pathway. Recent electroacupuncture studies have further confirmed that electroacupuncture at the Xinshu and Shenshu points can significantly enhance corticospinal excitability of the tibialis anterior, gastrocnemius, and vastus medialis muscles in the lower limb representation area of the motor cortex. The combined application of acupuncture and TEAS may produce a dual effect of “superimposed acupoint stimulation intensity” and “synergistic neural pathway activation”: acupuncture provides deep tissue stimulation, while TEAS provides continuous transcutaneous electrical stimulation, both acting on peripheral nerve terminals and converging onto the primary motor cortex (M1) via the spinal–cortical pathway, ultimately improving the output efficiency of the corticospinal tract more effectively. At the level of clinical evidence, the efficacy of acupuncture in improving EIF has been confirmed by meta-analysis. Niu et al.'s meta-analysis of 11 RCTs and 531 patients showed that acupuncture significantly alleviated subjective fatigue (SMD = −3.08, 95% CI: −4.35 to −1.81) and reduced blood lactate (SMD = −2.65, 95% CI: −3.52 to −1.78) and blood urea nitrogen (WMD = −1.47, 95% CI: −1.84 to −1.11) (Alix-Fages et al., 2019). On this basis, the present study further found that the effect of TEAS and acupuncture combined intervention was superior to that of acupuncture or TEAS alone, suggesting that the combined strategy has a potential “1 + 1 > 2” synergistic effect. This study conducted a network meta-regression to preliminarily explore the moderating effects of intervention type, total number of stimulations, sample size, and exercise modality on effect sizes. Results showed that the total number of stimulations negatively moderated improvements in blood lactate, suggesting that more stimulations are not necessarily better and may exhibit diminishing marginal returns. Sample size positively moderated blood lactate improvement, indicating that larger studies might report greater effects; however, this finding is limited by the small number of included studies and should be interpreted cautiously. Intervention type moderately influenced improvements in BUN and RPE, with central stimulation potentially outperforming peripheral stimulation in reducing BUN. Notably, exercise modality did not significantly moderate any outcomes, suggesting that despite differences in fatigue-inducing mechanisms across different exercises, the intervention effects observed in this study were relatively consistent across exercise types. However, due to the limited number of included studies and low statistical power, these meta-regression findings should be regarded strictly as exploratory insights for hypothesis generation rather than definitive conclusions. Future well-designed primary studies, particularly those with larger sample sizes and stratified by exercise type, are needed to validate these results.

### 4.3 Current status and development trends of neuromodulation techniques based on corticospinal excitability in treating athlete fatigue

In recent years, neuromodulation techniques based on corticospinal excitability modulation have made significant progress in the field of EIF intervention, but they also face challenges of inconsistent evidence and methodological heterogeneity. In terms of central stimulation techniques, tDCS is currently the most widely studied technique. Another meta-analysis, which included 14 RCTs and 376 athletes, confirmed that anodal tDCS significantly improved athletes' attention performance (*g* = 1.18) and cognitive function (*g* = 1.12), while reducing mental fatigue (*g* = −1.35) and reaction time (*g* = −1.35), indicating a significant effect of tDCS in modulating psychological functions in athletes (Mollazadeh and Ramezanpour, 2026). Other studies have confirmed that anodal tDCS stimulation of the M1 brain region can significantly reduce RPE during whole-body exercise and prolong time to exhaustion, but this effect has not been verified in single-joint resistance exercise (Alix-Fages et al., 2019). Holgado et al.'s umbrella review summarized 9 meta-analyses, 50 crossover studies, and 683 participants, finding that although the effect size of individual studies was significant, the effect size became non-significant after correcting for publication bias (Holgado et al., 2019). This study pointed out that the final effect size of different meta-analyses could vary from *g* = −0.23 to *g* = 0.33 due to differences in effect size calculation formulas and analytical procedures, indicating that the evidence for tDCS efficacy is not robust. A meta-analysis of patients with post-stroke fatigue also showed that non-invasive brain stimulation techniques could effectively relieve fatigue symptoms (Guan et al., 2023). TBS (theta burst stimulation), as an emerging TMS modality, has also shown potential for regulating EIF. Martignon et al. found that intermittent TBS can prolong exhaustion time, while continuous TBS can shorten exhaustion time due to aggravated central fatigue (Martignon et al., 2025), which explains the better performance of cTBS in improving RPE observed in this study. In terms of peripheral stimulation techniques, the evidence base for acupuncture and TEAS is more substantial. As mentioned previously, meta-analyses of acupuncture for improving EIF have confirmed its significant improvement effects on multiple fatigue indicators (Niu et al., 2022). On this basis, the network meta-analysis of the present study found that TEAS was superior to acupuncture alone, which may be related to the standardized and reproducible electrical stimulation parameters that TEAS can provide. In terms of development trends, future research may present the following directions: first, combined stimulation strategies will receive more attention; this study has already suggested the superiority of TEAS combined with acupuncture, and the synergistic effects of central and peripheral combined stimulation can be further explored. Second, individualized stimulation protocols will be a research focus; there is significant interindividual variability in current tDCS studies, which may be related to stimulation parameters, individual anatomical differences, and baseline corticospinal excitability levels. Third, long-term effects and safety need to be verified by more longitudinal studies. Fourth, standardized reporting needs to be strengthened to improve the comparability and synthesis of evidence.

Previous meta-analyses have mostly focused on the effects of a single central stimulation technique on motor performance, with controversial conclusions. Machado et al.'s meta-analysis included 14 studies and found that tDCS had a small to moderate positive effect on motor performance (Machado et al., 2019). Maudrich et al.'s meta-analysis focused on athletes, including 19 studies, and found that a single session of anodal tDCS significantly improved sport-specific performance in athletes (Maudrich et al., 2022). However, in Holgado et al.'s umbrella review, the effect size of tDCS was no longer significant after correcting for publication bias (Holgado et al., 2019). This inconsistency may be due to publication bias, differences in effect size calculation methods, and the inherent instability of tDCS effects. Niu et al.'s acupuncture meta-analysis included 11 RCTs and 531 patients, systematically confirming the efficacy of acupuncture in improving EIF (Niu et al., 2022). However, that study only conducted pairwise meta-analysis, failing to compare the relative efficacy of acupuncture with other neuromodulation techniques, and could not answer key clinical questions such as “whether acupuncture is superior to tDCS” or “whether acupuncture combined with TEAS is better.” In contrast, the present study used network meta-analysis, not only evaluating the efficacy of tDCS but also placing it within a broader network of neuromodulation techniques to provide more comprehensive evidence for efficacy ranking. In addition, the CINeMA evidence quality assessment in this study indicated that the evidence level for these comparisons was low, and the uncertainty of the available evidence may be greater than previously implied by other meta-analyses. The strengths and advances of this study are as follows: First, this study is the first to integrate central and peripheral neuromodulation techniques into the same network meta-analysis framework based on the common mechanism of corticospinal excitability. Second, it simultaneously evaluated three dimensions of fatigue subjective (RPE), metabolic (blood lactate), and protein breakdown (BUN). Third, meta-regression showed that the total number of stimulations had a negative moderating effect on BLA improvement (*P* = 0.001), and intervention type and study design significantly influenced BUN improvement. These findings provide important references for future research design.

### 4.6 Study strengths, limitations, and improvement measures

Strengths: First, this study used network meta-analysis for the first time to compare the effects of seven neuromodulation techniques on EIF within the same analytical framework, filling a research gap in this field. Second, based on the common neurophysiological mechanism of corticospinal excitability, this study provides a theoretical justification for the combined comparison of central and peripheral stimulation techniques. Third, meta-regression was used to explore the moderating effects of stimulation parameters, population characteristics, and study characteristics on effect size.

Limitations: First, the transitivity assumption was only partially satisfied. Stimulus timing and sport type varied considerably across studies, and both are key effect modifiers. This imbalance may compromise the validity of indirect comparisons. Although the diversity of sports and intervention protocols enhances external generalizability, it also introduces clinical heterogeneity. Therefore, the SUCRA rankings should be interpreted as exploratory hypotheses rather than definitive conclusions. Second, TEAS combined with acupuncture was defined as a single intervention node, and its effect size reflects the comprehensive efficacy of the complete regimen. The network cannot distinguish whether the observed benefit arises from synergistic effects or simply from a greater total stimulation dose. Factorial designs with dose-matching are needed to verify synergy, which were absent from the included studies. Accordingly, the interpretation of this combined node should be limited to its comprehensive efficacy, and mechanistic conclusions regarding synergy or superiority over single interventions should not be inferred. Third, several methodological limitations should be acknowledged: (1) stimulation parameters (intensity, duration, frequency, acupoint selection) varied across studies, introducing clinical heterogeneity; (2) some studies used sham stimulation/sham acupuncture while others used blank control, and these conditions are not physiologically equivalent, which may have diluted or exaggerated effect estimates; (3) Outcome measurement time points for RPE, BLA, and BUN differed across studies; (4) 10 studies were included per outcome, publication bias tests, sensitivity analyses, and subgroup analyses could not be conducted with adequate statistical power. Although meta-regression was used to explore covariates, the small number of studies limited statistical power, and the aggregate-data approach precludes individual-level validation. Fourth, the primary outcomes analyzed are indirect indicators of exercise-induced fatigue rather than direct measures of corticospinal excitability. The CSE-based framework served as a conceptual rationale for integrating central and peripheral neuromodulation techniques, but CSE changes were not directly measured in the included trials. The mechanistic interpretation of our findings should therefore be considered hypothesis-generating rather than confirmatory. Future studies should incorporate direct CSE measurements as primary outcomes. Fifth, the external validity and generalizability of our findings are limited by the geographic and ethnic composition of the included studies. All 14 RCTs were conducted in Asia, with samples predominantly consisting of Chinese athletes. Population genetics may influence responses to tDCS and acupuncture; cultural factors may shape treatment expectations and placebo responses, particularly for acupuncture and TEAS; and dietary, training, and environmental differences may further moderate intervention effects. These factors collectively limit the applicability of our conclusions to non-Chinese athlete populations. Future multi-center, multi-ethnic studies with stratification by genetic and cultural factors are needed to establish cross-population validity.

Improvement Measures: First, future studies should standardize and clearly report stimulus timing, and stratify or adjust for sport type, to improve the validity of indirect comparisons in future network meta-analyses. Second, factorial designs with dose-matched comparisons are needed to isolate synergistic effects from additive dose effects. Third, future original studies should uniformly report stimulation parameters and outcome measurement time points, standardize sham procedures, and include both sham and blank control arms where possible. Once sufficient studies accumulate, sensitivity, subgroup, and dose-response analyses should be performed with adequate statistical power, and individual participant data meta-analysis should be considered. Fourth, future studies should incorporate direct measures of corticospinal excitability as primary outcomes to validate the proposed mechanism. Fifth, future research should be conducted in multi-center, multi-ethnic settings with stratification by genetic and cultural factors, and should prioritize larger-sample RCTs targeting TEAS combined with acupuncture and cTBS.

## 5 Conclusions

This study, through a systematic review and network meta-analysis, comprehensively compared the efficacy of neuromodulation techniques in alleviating athletic fatigue for the first time, based on corticospinal excitability as a common target. Pairwise meta-analyses confirmed that neuromodulation interventions significantly reduced athletes' RPE, BLA, and BUN levels. The network meta-analysis revealed that TEAS combined with acupuncture demonstrated the highest SUCRA ranking across all three core outcome measures. However, the evidence quality for all main findings was low, and most comparisons were based on single studies; thus, the SUCRA rankings should be regarded as exploratory rather than definitive conclusions. Future research should conduct more high-quality, large-sample randomized controlled trials to validate these preliminary findings and systematically explore optimal stimulation parameters to advance the precise application of neuromodulation techniques in managing exercise-induced fatigue.

## Data Availability

The original contributions presented in the study are included in the article/Supplementary material, further inquiries can be directed to the corresponding author.
