# Supplementary material for: Comparison of the efficacy of neuromodulation techniques based on corticospinal excitability in improving exercise-induced fatigue in athletes: a systematic review and network meta-analysis
Source: Front Psychol. 2026 Oct 13;17:1948789. doi: 10.3389/fpsyg.2026.1948789 (PMC13710033; doi:10.3389/fpsyg.2026.1948789)
Supplement: Supplementary file 1 [file Table_1.docx]

**1. Search Strategy**

(1)PubMed,EMBASE,Cochrane library

| **Search number** | **Query** |
| --- | --- |
| #1 | Athletes OR Sports[MeSH Terms] |
| #2 | Athlete OR Sport* OR Athletic*[Title/Abstract] |
| #3 | #1 OR #2 |
| #4 | Transcranial Direct Current Stimulation OR Transcranial Magnetic Stimulation OR Acupuncture OR Acupuncture Therapy OR Acupuncture, Ear[MeSH Terms] |
| #5 | Transcranial Direct Current Stimulation OR Transcranial Alternating Current Stimulation OR Transcranial Random Noise Stimulation OR Transcranial Electrical Stimulation* OR Transcranial Magnetic Stimulation* OR transcutaneous acupoint electrical stimulation OR tDCS* OR tACS OR tRNS OR TMS OR rTMS OR transcranial electric* OR brain stimulation OR Acupuncture OR Pharmacopuncture OR Electroacupuncture [Title/Abstract] |
| #6 | #4 OR #5 |
| #7 | Muscle Fatigue[MeSH Terms] |
| #8 | exercise-induced fatigue OR central fatigue OR peripheral fatigue OR Muscle Fatigue OR Fatigue[Title/Abstract] |
| #9 | #7 OR #8 |
| #10 | Randomised OR Randomized[Title/Abstract] |
| #11 | #6 AND #9 AND #10 |

(2)Web of science,CNKI,VIP,Wanfang,SinoMed

| **Search number** | **Query** |
| --- | --- |
| #1 | Athlete OR Sport* OR Athletic*[Title/Abstract] |
| #2 | Transcranial Direct Current Stimulation OR Transcranial Alternating Current Stimulation OR Transcranial Random Noise Stimulation OR Transcranial Electrical Stimulation* OR Transcranial Magnetic Stimulation* OR transcutaneous acupoint electrical stimulation OR tDCS* OR tACS OR tRNS OR TMS OR rTMS OR transcranial electric* OR brain stimulation OR Acupuncture OR Pharmacopuncture OR Electroacupuncture [Title/Abstract] |
| #3 | exercise-induced fatigue OR central fatigue OR peripheral fatigue OR Muscle Fatigue OR Fatigue [Title/Abstract] |
| #4 | Randomised OR Randomized[Title/Abstract] |
| #5 | #1 AND #2 AND #3 AND #4 |

**2.League Table**

(1)RPE

| AMT | Auricularpressure | cTBS | Placebo | tDCS | TEAS | TEASandAcupuncture | TEAS(no electricity) |
| --- | --- | --- | --- | --- | --- | --- | --- |
| AMT | -3.78 (-6.48, -1) | -4.62 (-7.84, -1.35) | 1.2 (-0.74, 3.18) | 1.31 (-1.06, 3.71) | -5.18 (-7.48, -2.75) | -9.51 (-12.76, -6.13) | -4.64 (-7.18, -1.96) |
| 3.78 (1, 6.48) | Auricularpressure | -0.82 (-4.03, 2.3) | 4.98 (3.05, 6.84) | 5.09 (2.69, 7.39) | -1.4 (-3.7, 0.94) | -5.71 (-8.99, -2.43) | -0.86 (-3.41, 1.74) |
| 4.62 (1.35, 7.84) | 0.82 (-2.3, 4.03) | cTBS | 5.8 (3.22, 8.45) | 5.9 (3, 8.84) | -0.57 (-3.39, 2.37) | -4.87 (-8.59, -1.14) | -0.03 (-3.07, 3.07) |
| -1.2 (-3.18, 0.74) | -4.98 (-6.84, -3.05) | -5.8 (-8.45, -3.22) | Placebo | 0.11 (-1.28, 1.45) | -6.38 (-7.66, -5.01) | -10.7 (-13.39, -7.98) | -5.84 (-7.54, -4.03) |
| -1.31 (-3.71, 1.06) | -5.09 (-7.39, -2.69) | -5.9 (-8.84, -3) | -0.11 (-1.45, 1.28) | tDCS | -6.49 (-8.32, -4.54) | -10.81 (-13.78, -7.76) | -5.95 (-8.07, -3.69) |
| 5.18 (2.75, 7.48) | 1.4 (-0.94, 3.7) | 0.57 (-2.37, 3.39) | 6.38 (5.01, 7.66) | 6.49 (4.54, 8.32) | TEAS | -4.33 (-6.77, -1.9) | 0.54 (-1.2, 2.29) |
| 9.51 (6.13, 12.76) | 5.71 (2.43, 8.99) | 4.87 (1.14, 8.59) | 10.7 (7.98, 13.39) | 10.81 (7.76, 13.78) | 4.33 (1.9, 6.77) | TEASandAcupuncture | 4.86 (1.99, 7.81) |
| 4.64 (1.96, 7.18) | 0.86 (-1.74, 3.41) | 0.03 (-3.07, 3.07) | 5.84 (4.03, 7.54) | 5.95 (3.69, 8.07) | -0.54 (-2.29, 1.2) | -4.86 (-7.81, -1.99) | TEAS(no electricity) |

(2)BLA

| Acupuncture | Placebo | tDCS | TEAS | TEASandAcupuncture |
| --- | --- | --- | --- | --- |
| Acupuncture | 0.48 (-0.42, 1.33) | -0.18 (-2.03, 1.64) | -1.56 (-3.24, 0.05) | -2.51 (-4.82, -0.25) |
| -0.48 (-1.33, 0.42) | Placebo | -0.66 (-2.27, 0.97) | -2.05 (-3.44, -0.66) | -2.99 (-5.1, -0.9) |
| 0.18 (-1.64, 2.03) | 0.66 (-0.97, 2.27) | tDCS | -1.39 (-3.52, 0.71) | -2.33 (-4.99, 0.31) |
| 1.56 (-0.05, 3.24) | 2.05 (0.66, 3.44) | 1.39 (-0.71, 3.52) | TEAS | -0.94 (-2.54, 0.65) |
| 2.51 (0.25, 4.82) | 2.99 (0.9, 5.1) | 2.33 (-0.31, 4.99) | 0.94 (-0.65, 2.54) | TEASandAcupuncture |

(3)BUN

| Acupuncture | AMT | Auricularpressure | Placebo | TEAS | TEASandAcupuncture | TEAS(no electricity) |
| --- | --- | --- | --- | --- | --- | --- |
| Acupuncture | -0.85 (-7.37, 5.72) | 0.51 (-6.04, 7.03) | 1.44 (-3.2, 6.05) | -0.67 (-6.27, 5.07) | -1.73 (-9.03, 5.7) | -0.59 (-6.19, 5.16) |
| 0.85 (-5.72, 7.37) | AMT | 1.35 (-5.2, 7.91) | 2.29 (-2.42, 6.94) | 0.17 (-5.47, 5.98) | -0.88 (-8.19, 6.59) | 0.25 (-5.39, 6.02) |
| -0.51 (-7.03, 6.04) | -1.35 (-7.91, 5.2) | Auricularpressure | 0.93 (-3.67, 5.51) | -1.18 (-6.8, 4.56) | -2.24 (-9.56, 5.2) | -1.1 (-6.71, 4.63) |
| -1.44 (-6.05, 3.2) | -2.29 (-6.94, 2.42) | -0.93 (-5.51, 3.67) | Placebo | -2.11 (-5.35, 1.27) | -3.17 (-8.82, 2.66) | -2.03 (-5.25, 1.37) |
| 0.67 (-5.07, 6.27) | -0.17 (-5.98, 5.47) | 1.18 (-4.56, 6.8) | 2.11 (-1.27, 5.35) | TEAS | -1.06 (-5.73, 3.58) | 0.08 (-3.22, 3.39) |
| 1.73 (-5.7, 9.03) | 0.88 (-6.59, 8.19) | 2.24 (-5.2, 9.56) | 3.17 (-2.66, 8.82) | 1.06 (-3.58, 5.73) | TEASandAcupuncture | 1.14 (-4.58, 6.9) |
| 0.59 (-5.16, 6.19) | -0.25 (-6.02, 5.39) | 1.1 (-4.63, 6.71) | 2.03 (-1.37, 5.25) | -0.08 (-3.39, 3.22) | -1.14 (-6.9, 4.58) | TEAS(no electricity) |

**3.Meta Regression**

|  | RPE | BL | BUN |
| --- | --- | --- | --- |
| Acting path | 0.06 | 0.64 | 0.03 |
| Single duration(min) | 0.11 | 0.91 | 0.4 |
| Number of treatments | 0.1 | 0.001 | 0.36 |
| Age | 0.06 | 0.51 | 0.08 |
| Study design | 0.92 | 0.3 | 0.03 |
| Risk | 0.13 | 0.91 | 0.02 |
| Year | 0.01 | 0.68 | 0.12 |
| Sample size | 0.30 | 0.01 | 0.76 |
| Type of sports | 0.42 | 0.21 | 0.65 |
